# Relationship between Tertiary Lymphoid Structure and the Prognosis and Clinicopathologic Characteristics in Solid Tumors

**DOI:** 10.7150/ijms.56347

**Published:** 2021-04-07

**Authors:** Zhan Zhao, Hui Ding, Zheng-bin Lin, Sheng-hui Qiu, Yi-ran Zhang, Yan-guan Guo, Xiao-dong Chu, Loi I Sam, Jing-hua Pan, Yun-long Pan

**Affiliations:** 1 Department of General Surgery, the First Affiliated Hospital of Jinan University, Guangzhou 510632, China.; 2 International School, Jinan University, Guangzhou 510632, China.

**Keywords:** tertiary lymphoid structure (TLS), tumor, overall survival, disease-free survival, relapse-free survival, clinicopathologic characteristics.

## Abstract

**Background:** An increasing number of studies had shown that tertiary lymphoid structure (TLS) plays an important role in tumor progression. However, the prognostic role of TLS in various tumors remains controversial. This meta-analysis aims to investigate the clinicopathological and prognostic values of TLS in solid tumors.

**Methods:** A systematic search was conducted in PubMed, EMBASE and Cochrane Library undated to November 2, 2020. Odds ratios of clinical parameters, hazard ratio (HR) of overall survival (OS), relapse-free survival (RFS), disease-free survival (DFS) and relapse rate were calculated in order to evaluate the relationship between TLS expression and clinicopathological or prognostic values in different tumors.

**Result:** 27 eligible studies including 6647 patients with different types of tumors were analyzed. High TLS expression was associated with a longer OS (HR = 0.66, 95% CI: 0.50 - 0.86, *P* = 0.002) and RFS (HR = 0.61, 95% CI: 0.47 - 0.79, *P* = 0.0001). Moreover, high TLS levels in tumor were associated with a low risk of recurrence (HR = 0.43, 95% CI: 0.32 - 0.57, *P* < 0.0001). However, there was no relationship between TLS expression and DFS. Meanwhile, high TLS expression was associated with smaller tumor size (*P* < 0.00001) and higher tumor infiltrating lymphocytes (TILs). Furthermore, the subgroup analysis showed high TLS expression that may be associated with a lower clinical grading and N stage in breast cancer and colorectal cancer.

**Conclusion:** High TLS expression is associated with the longer OS and RFS in solid tumors, and a lower risk of cancer relapse. Meanwhile, high TLS expression is also associated with a smaller tumor size, higher infiltration of TILs, lower clinical grading and N stage in the tumor. Therefore, high TLS expression in the tumor is a favorable prognostic biomarker for solid tumor patients.

## Introduction

Worldwide data suggests that the incidence and mortality of cancer are rapidly increasing over the past decades. Cancer is expected to be the leading cause of death and one of the major obstacles to prolong life expectancy 1. Conventional therapies including radiotherapy and chemotherapy have limited therapeutic effects. Immunotherapy, a novel strategy for cancer treatment, has achieved significant success 2. However, the limited numbers of biomarkers are difficult to predict and evaluate the therapeutic response of immunotherapy. Although programmed cell death-ligand 1 (PD-L1), deficient mismatch repair (dMMR) / microsatellite instability -high (MSI-H) and tumor mutational burden (TMB) had been considered as immunotherapy biomarkers. However, exploring more precise biomarkers is still a research focus for immunotherapy.

Tertiary lymphoid structure (TLS) is a crucial element of the tumor immune microenvironment (TIME), which consists of T cells, B cells, fibroblastic reticular cell (FRC) network, high endothelial venules (HEVs) and follicular dendritic cells (FDCs) 3-6. Recently, some studies had proved that TLS play an important role in different kinds of tumor 7-9. TLS has been shown to improve the effect of immunotherapy and patient survival in various tumors due to its relation with immune cell infiltration in melanoma 10, 11. On the one hand, peritumoral TLS expression indicates unfavorable clinicopathological characteristics and a worse prognosis in hepatocellular carcinoma 12-14. However, some studies also reported no association between TLS expression and overall survival in breast cancer 9, 15. Therefore, the relationship between TLS expression and clinicopathological characteristics and prognosis of the tumor still remains controversial.

This meta-analysis aims to investigate the clinicopathological characteristics and prognostic value of TLS expression in solid tumors based on published research.

## Materials and Methods

### Search identification

PubMed, EMBASE and Cochrane library was searched up to November 15, 2020 for primary studies, focusing on the relationship between TLS and human colorectal cancer. We designed a strategy consisting of Medical Subject Headings (MeSH), common keywords and their comprehensive combination to strengthen the sensitivity of the search. The following MeSH and common keywords were included: 'Tertiary Lymphoid Structure', 'Lymphoid Structures, Tertiary', 'Tertiary Lymphoid Organ', 'Ectopic Lymphoid Like Structure', 'Ectopic Lymphoid Organ', 'Tertiary Lymphoid Tissue', 'Ectopic Lymphoid Follicle', 'Ectopic Lymphoid Formations', 'Neoplasms', 'Tumors', 'Neoplasia', 'Cancer', 'Malignancy'. Randomly combing the MeSH terminology and relevant keywords ensures that the most comprehensive data were acquired. No language restriction was applied.

### Inclusion and exclusion criteria

All eligible studies were initially screened by two authors (Z.Z and H.D) based on the title and abstract, then those considered potentially relevant were retrieved for full-text review. Any disagreement was resolved by discussion with a third author (J.P). The inclusion criteria were as follows: (1) Studies focused on patients with solid tumor and TLS expression; (2) Differential expressions of TLS in cancer tissue compared to adjacent non-cancerous tissue must be presented; (3) TLS were measured by a standard method; (4) The relationship between TLS expression and its clinical outcome with hazard ratios (HRs) of 95% CI from each study could be extracted; (5) Studies included relationship between TLS expression and clinicopathological characteristics.

The exclusion criteria were as follows: (1) case reports, reviews, literature interpretations, personal view, grey literature; (2) The same data has been used in other studies; (3) Lack of clinical features and prognostic data.

### Data extraction

According to PRISMA, two researchers (Z.Z and H.D) extracted data from all the included literature independently and any disagreement was resolved by a consensus with the third author (J.P) 16, 17. The following data were extracted: first author, year of publication, country, types of tumors, number of patients, gender, age, expressions of Ki67, Cut-off criteria, TMN stage, clinical stage, detection of TLS, survival analysis, follow-up time, cut-off criteria; prognostic outcomes including HRs of high TLS expression for overall survival (OS), relapse-free survival (RFS), disease-free survival (DFS), relapse rate. If the literature just provided Kaplan-Meier curves, we estimated the statistics by Engauge Digitizer version 4.1 and utilize the spreadsheet developed by Jayne F Tierney for data extraction 18, 19 (**Table 1** and **Table 2**).

### Quality assessment

The above studies were evaluated independently by two researchers (L.Z and X.C) using the Newcastle-Ottawa Scale (NOS) 20. The score was calculated based on three factors: selection, comparability and outcome, with a full score of 9. The included studies with a score greater than 6 is considered as high quality 19. The quality of the included studies in the current meta-analysis was high, with a score of not less than 6 per article (**Table 1**) 7-9, 12, 13, 15, 21-41.

### Statistical analysis

In our research, the prognostic value of TLS expression in patients with various tumors was assessed by the pooled HRs and its relevant 95% CIs. A pooled HRs > 1 implied a worse survival with high expressions of TLS, while HRs < 1 indicated a favorable outcome. Odds ratio (OR) and the corresponding 95% CIs were used to analyze the association between TLS expression and its clinicopathological characteristics. In addition, the heterogeneity between literature with *P*-value and *I^2^* were estimated. If *I^2^* > 50% or *P* < 0.1, it is believed that the studies exhibit obvious heterogeneity and a random-effect model was applied. Otherwise, if *I^2^* < 50% or *P* > 0.1, fix-effects model was used. Subgroup analysis was then performed to investigate the source of heterogeneity. Subsequently, funnel plot was conducted to detect the publication bias. *P* < 0.05 was considered statistically significant in all tests. All statistical analysis was done by Review Manager 5.3.

## Results

**Figure 1:**
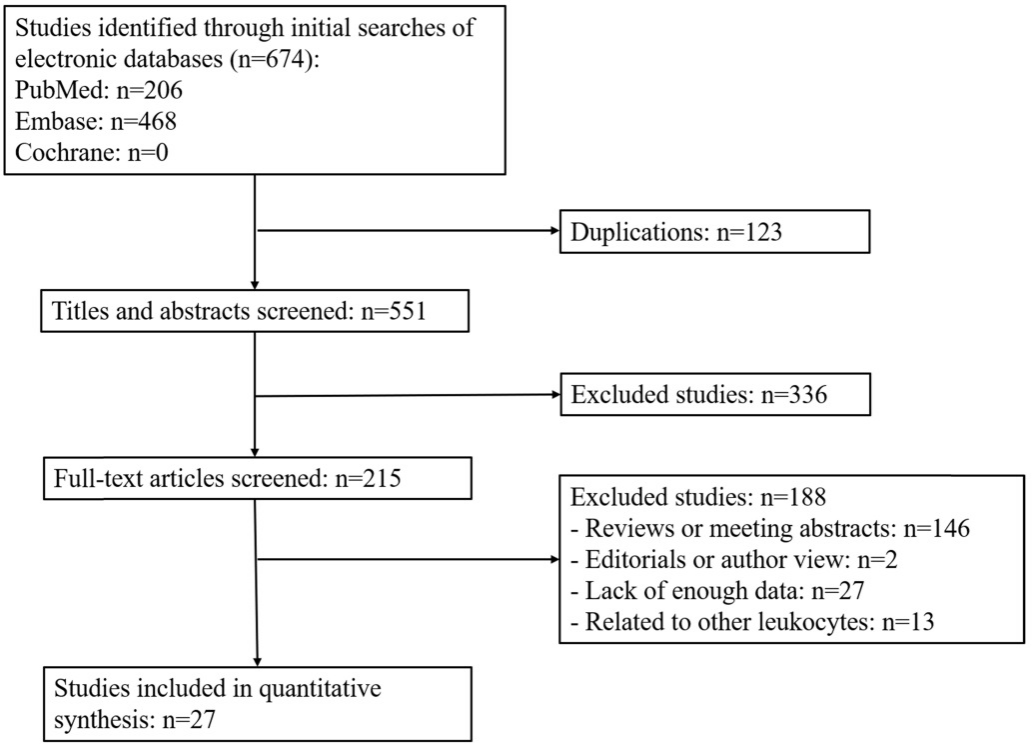
Flow diagram of study selection process.

Based on the above retrieval strategy, we initially identified 674 articles from the databases according to the keywords and excluded 123 duplicated articles. Among them, 336 articles were discarded after reviewing their abstracts or titles. The remaining articles were reviewed based on our inclusion criteria. Among the 215 articles, 146 were reviews or conference abstracts, so no relevant clinical data could be extracted; 27 articles did not provide enough data to generate relevant results; 13 articles were related to other leukocytes rather than TLS; 2 articles were author views or editorials. Finally, 27 eligible studies (n = 6647 patients) were included in the current meta-analyses (**Figure 1**).

### Study characteristics

The characteristics of the included articles are presented in **Table 1**. In our analysis, there were 5 studies focusing on colorectal cancer (CRC), 8 on breast cancer (BC), 2 on hepatocellular carcinoma (HCC), 3 on pancreatic tumor, 3 on gastric tumor (GT), 3 on oral squamous cell carcinoma (OSCC), 1 on esophageal squamous cell carcinoma (ESCC), Merkel cell carcinoma and lung squamous cell carcinoma (SCC) respectively. Clinical outcomes were assessed in 15 studies. Among them, 17 studies including 4748 patients used OS as the primary endpoint. 5 studies, including 1046 patients used relapse as the primary endpoint. 7 studies, including 1855 patients reported data on DFS. 4 studies, including 994 patients took RFS as the endpoint. The expressions of TLS were detected using the same method (immunohistochemistry) in most articles. Besides, we found that there were 5 studies with two different independent cohorts, which were included separately as independent cohorts for further statistics 8, 9, 25, 34, 41.

### Association between TLS expression and prognosis of solid tumor patients

**Figure 2:**
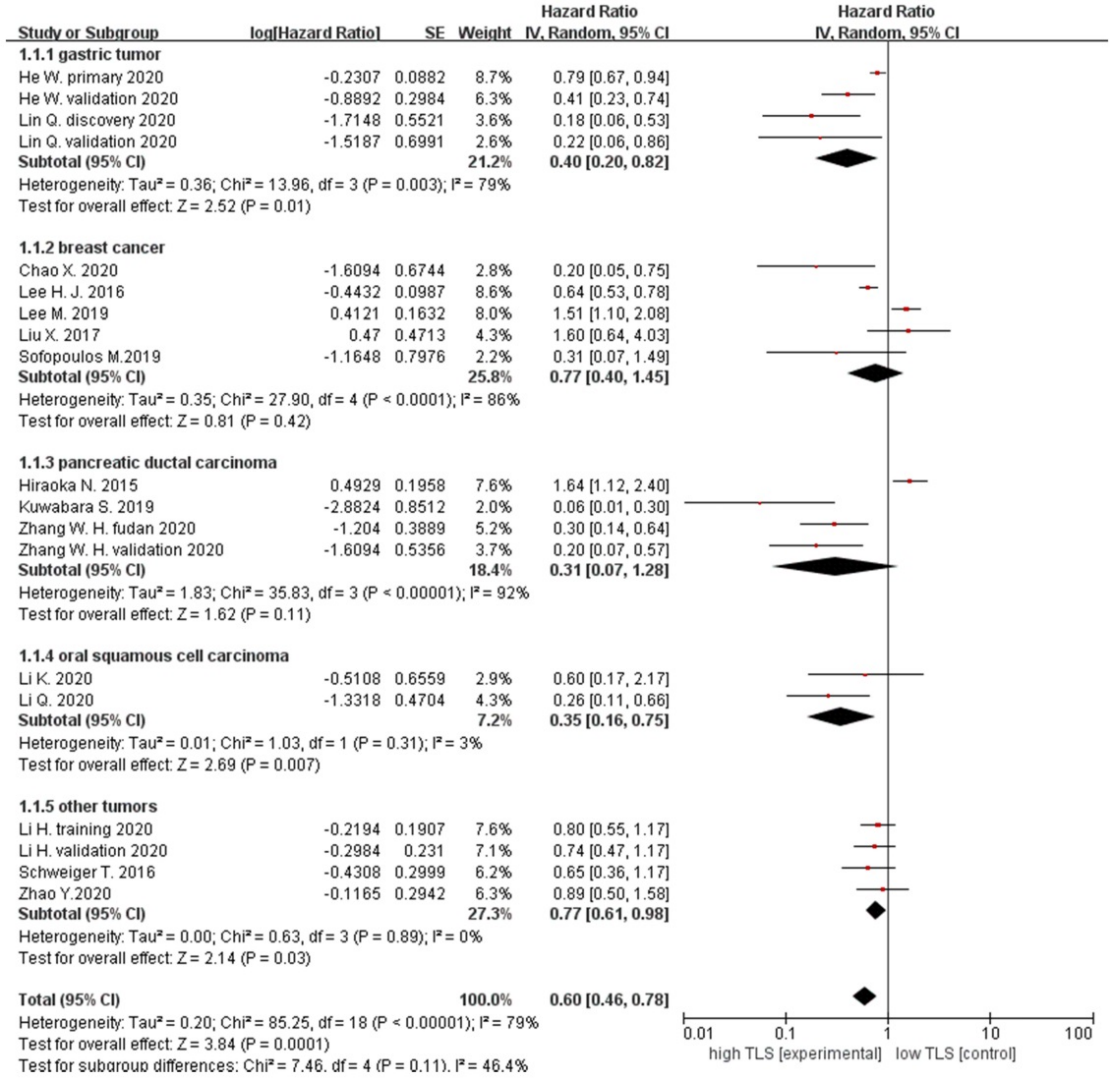
Forest plot for OS outcomes for different types of the tumor with TLS expression.

We analyzed TLS expression within different types of cancer in 17 studies. Firstly, patient survival was evaluated by OS. The results showed that the pooled HRs of all patients with cancer was 0.60 (95% CI: 0.46 - 0.78, *P* = 0.0001), suggesting that high TLS expression was correlated with longer OS in solid tumors. Furthermore, to assess the relationship between TLS expression and prognosis in a different type of tumors, the data were divided into five subgroups according to the tumor types. The pooled HRs for breast cancer OS was 0.77 (95% CI: 0.40 - 1.45, *P* = 0.42), gastric cancer was 0.40 (95% CI: 0.20 - 0.82, *P* = 0.01) and 0.31 for pancreatic cancer (95% CI: 0.07 - 1.28, *P* = 0.11). For oral squamous cell carcinoma, OS was 0.35 (95% CI: 0.16 - 0.75, *P* = 0.007) and 0.77 (95% CI: 0.61 - 0.98, *P* = 0.03) for other cancer. Therefore, our results demonstrated that high TLS expression is correlated with better prognosis with GT and OSCC. However, subgroup analysis also indicated no correlation between TLS expression and OS in BC and pancreatic tumors (**Figure 2**).

**Figure 3:**
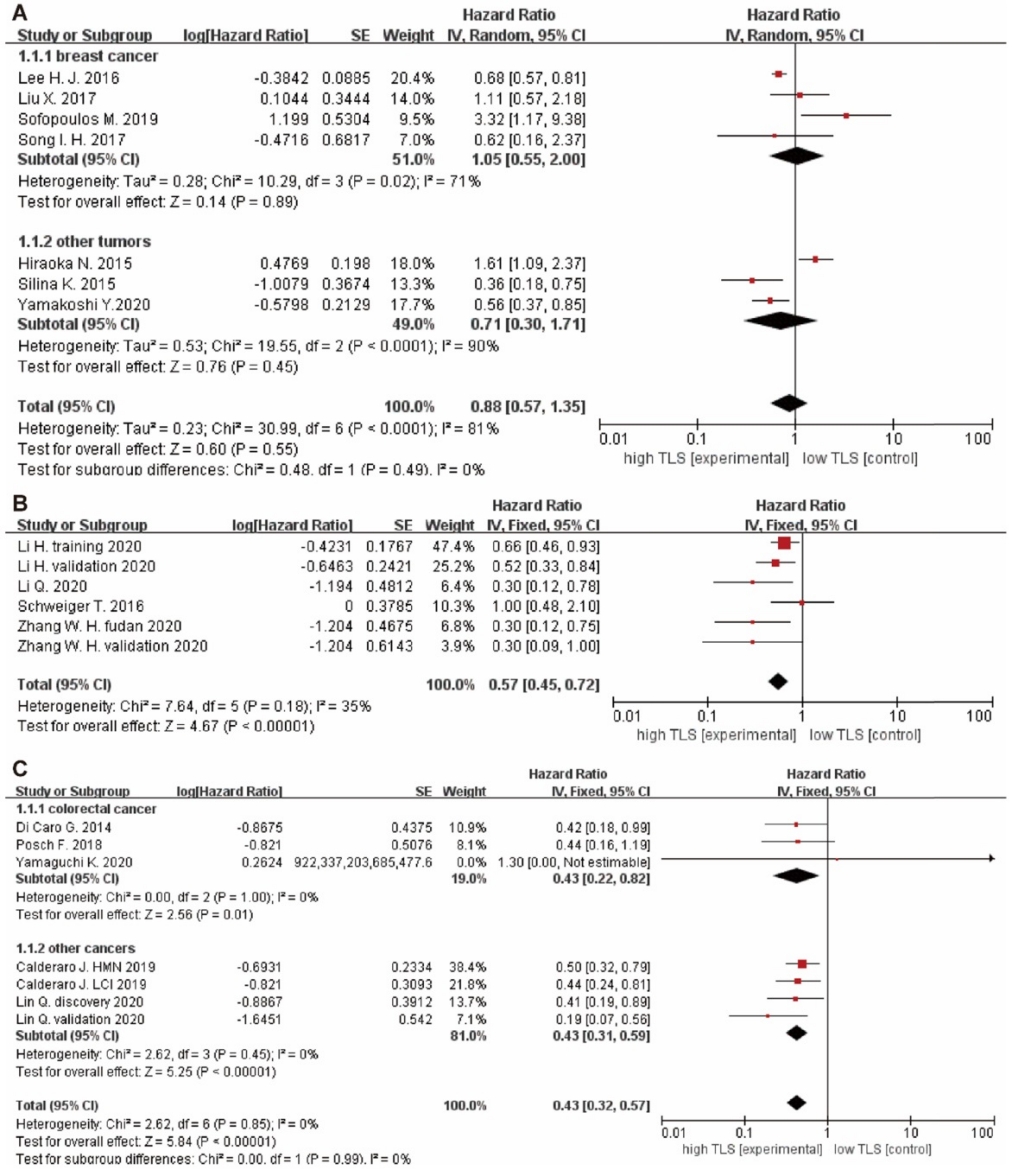
** Forest plot for DFS, RFS and relapse rate analysis.** (A) DFS outcome of cancer with high TLS expression versus low TLS expression. (B) RFS outcome of cancer with high TLS expression versus low TLS expression. (C) Relapse rate outcome of cancer with high TLS expression versus low TLS expression. Each result is shown by the HR with 95%.

Moreover, we also evaluated the role of TLS in DFS of solid patients. 7 studies with DFS data were showed that HRs in all tumor patients was 0.88 (95% CI: 0.57 - 1.35, *P* = 0.55), indicating that there was no relationship between TLS expression and DFS in tumor patients, especially in the breast cancer subgroup (HR = 1.05, 95% CI: 0.55 - 2.00, *P* = 0.89) (**Figure 3A**). In addition, 4 studies focusing on RFS has a pooled HR of 0.57 (95% CI: 0.45 - 0.72, *P* < 0.0001), which suggested that high TLS expression in the tumor were correlated with longer RFS compared with low or negative TLS expression in patients with tumor (**Figure 3B**).

For the correlation between TLS expression and relapse rate of tumor, 5 studies including 821 patients were selected for analysis. The results showed that the pooled HRs of relapse rate in all patients were 0.43 (95% CI: 0.32 - 0.57, *P* < 0.0001), suggesting that high TLS expression was correlated with lower risk of recurrence compared with low or negative TLS expression in tumor patients. Subgroup analysis showed high TLS expression was correlated with lower relapse rate in CRC (HRs = 0.43, 95% CI: 0.22 - 0.82, *P* = 0.01) and other cancers (HRs = 0.43, 95% CI: 0.31 - 0.59, *P* < 0.00001) (**Figure 3C**).

### Association between TLS expression and clinical characteristics in solid tumors

**Figure 4:**
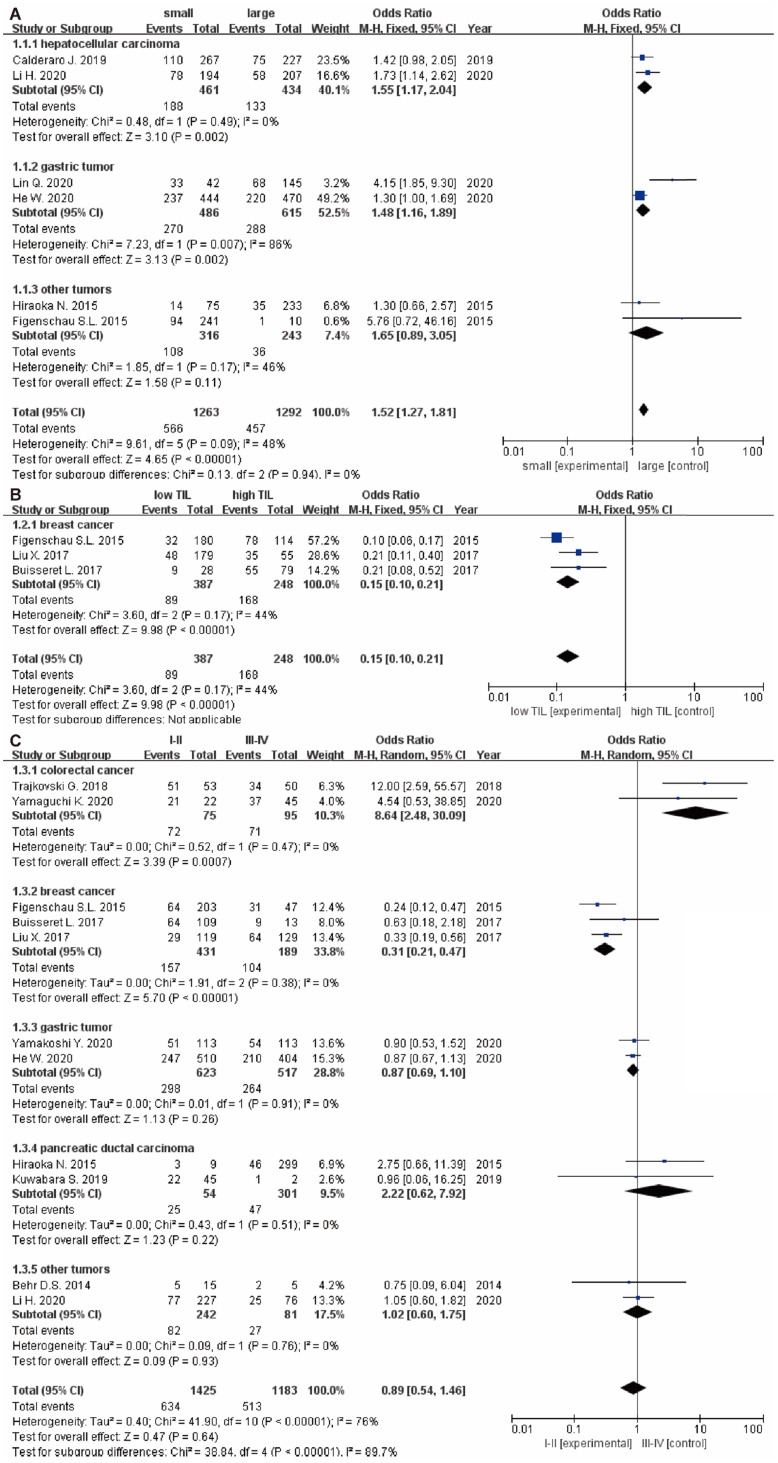
** The forest plot of OR was assessed for association between TLS and clinicopathological characteristics.** (A) tumor size; (B) TILs; (C) grade. Each result is shown by the OR with 95% CI.

The relationship between TLS expression and clinicopathological characteristics are illustrated in **Table 2**. 11 studies 9, 15, 22-24, 29-32, 34, 35 displayed original data on the relationship between TLS expression and clinicopathological characteristics. For tumor growth, the pooled results showed that high TLS expression was significantly associated with smaller tumor size in overall solid tumors (OR: 1.52, 95% CI: 1.27 - 1.81, *P* < 0.00001), especially in the HCC (OR: 1.55, 95% CI: 1.17 - 2.04, *P* = 0.002) and GC respectively (OR: 1.48, 95% CI: 1.16 - 1.89, *P* = 0.002) (**Figure 4A**). Moreover, low TLS expression in clinicopathological tissue were significantly associated with lower TILs in tumor (OR: 0.15, 95% CI: 0.10 - 0.21, *P* < 0.00001) (**Figure 4B**).

**Figure 5:**
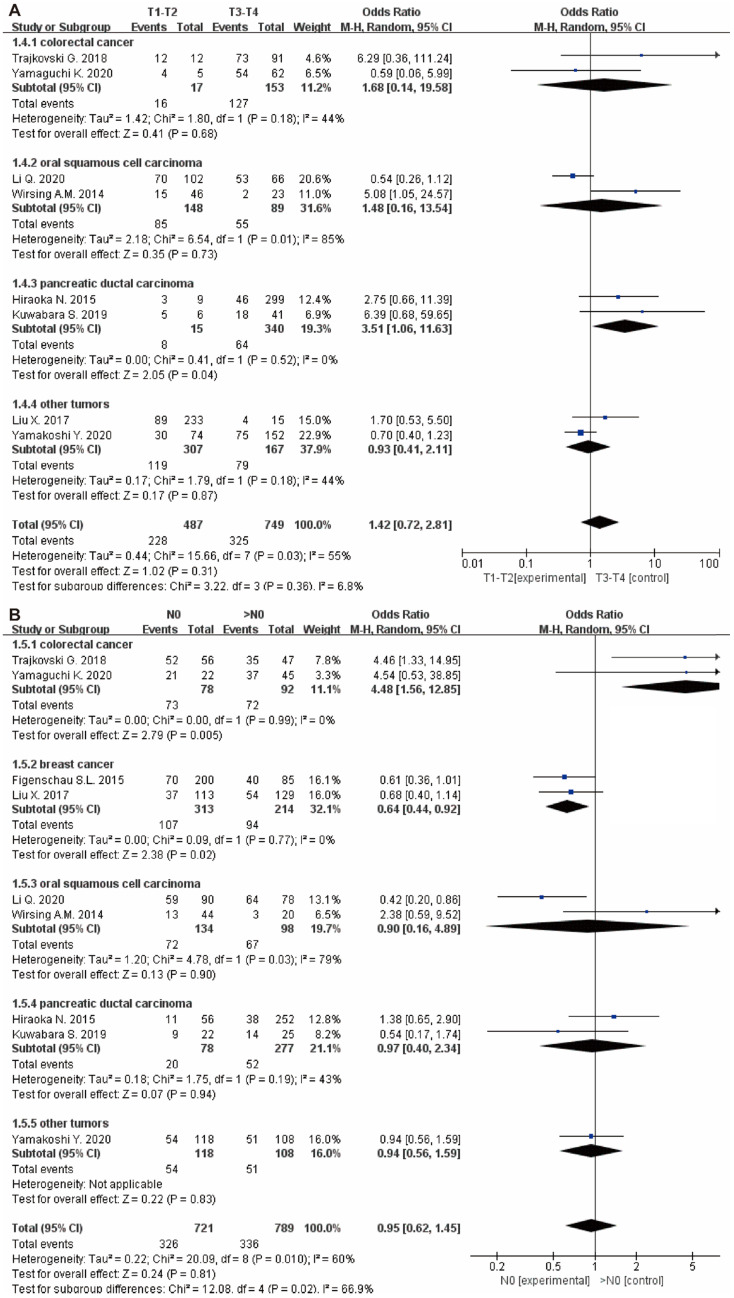
** Forest plot of the association between TLS expression with clinicopathological characteristics.** (A): T stage; (B): N stage.

Besides, there were no significant correlation between TLS expression and the T stage (OR: 1.42, 95% CI: 0.72 - 2.81, *P* = 0.31) (**Figure 5A**), N stage (OR: 0.95, 95% CI: 0.62 - 1.45, *P* = 0.81) (**Figure 5B**) and clinical grade (OR: 0.89, 95% CI: 0.54 - 1.46, *P* = 0.64) (**Figure 4C**). However, high TLS expression was associated with a lower clinical grade and a lower N stage in the colorectal cancer and breast cancer subgroup. Regarding the T stage, higher TLS expression had a positive correlation with a lower T stage in the pancreatic cancer subgroup.

Finally, TLS expression were considered to have no relationship with the patient age (OR: 0.91, 95% CI: 0.76 - 1.11, *P* = 0.36) (**Supplementary Figure 1A**), gender (OR: 1.11, 95% CI: 0.87 - 1.41, *P* = 0.40) (**Supplementary Figure 1B**), expressions of ki67 (OR: 0.71, 95% CI: 0.29 - 1.75, *P* = 0.46) (**Supplementary Figure 1C**) and M stage (OR: 0.49, 95% CI: 0.17 - 1.38, *P* = 0.18) (**Supplementary Figure 1D**).

### Publication Bias

We assessed the publication bias by funnel plots, as shown in Figure 4. There was no obvious publication bias for OS (**Supplementary Figure 2A**), DFS (**Supplementary Figure 2B**), RFS (**Supplementary Figure 2C**) and clinicopathological characteristics (**Supplementary Figure 3**).

## Discussion

The crosstalk between tumor cells and tumor immune system has gained people attention for cancer immunotherapy including monoclonal antibodies, cancer vaccines and adoptive T cell therapy 42-44. A large number of studies demonstrated that the tumor microenvironment is closely related to the prognosis and effectiveness of immunotherapy 45-47. TLS is a crucial component of tumor immune microenvironment 5, 48, which not only facilitating the recruitment of immune cells or play an anti-tumor role, but also act as a predictor for the prognosis of various cancer 4, 7, 10, 15, 22, 24, 34, 39. However, several studies reported the effect of TLS expression on prognosis and clinicopathological characteristics of solid tumor patient remains controversial 9, 11, 13, 15. To the best of our knowledge, this is the first comprehensive meta-analysis on the prognostic value and clinicopathological characteristics of TLS in various solid tumors. We provided strong evidence that high expression of TLS in tumor show a favorable prognostic value of tumor patients in terms of OS, RFS and relapse rate. Additionally, TLS expression is associated with tumor grade (especially in colorectal cancer and breast cancer), T stage (mainly in pancreatic cancer), N stage (particularly in colorectal cancer and breast cancer), tumor size (mostly in liver cancer and gastric cancer) and TILs (especially in breast cancer).

Firstly, the prognostic value of TLS in tumor patients is evaluated systemically. Our finding revealed no significant correlation between the high expressions of TLS and DFS in tumor patients. However, patients with higher TLS expression have a longer OS, RFS and lower relapse rate. In the subgroup analysis of OS in patients with gastric cancer and oral squamous cell carcinoma, patients with a higher TLS expression obviously have longer OS than those with a lower TLS expression. Nonetheless, the results in the breast cancer and pancreatic cancer subgroup showed that TLS expression has no relationship with OS. Using the same method to access the results of the DFS and relapse rate, no obvious correlation was obtained in the DFS subgroup. In the relapse subgroup, colorectal cancer patients with a higher TLS expression showed a lower risk of relapse. Therefore, we conclude that TLS expression is a prognostic biomarker for tumor patients in terms of OS and RFS, but not DFS. Meanwhile, TLS is also a biomarker for recurrence rate in tumor patients. Previous studies have found that the gene expression of TLS confirmed a prognostic role in melanoma, which are correlated with the gene of B cell, T cell, other types of immune cell and RNA-seq data for metastatic melanomas 49-52. Meanwhile, the gene expression analysis of TLSs identified pathways regulating immune cell activation and trafficking, a suppressed regulatory T (Treg) cell induction pathway and an enhanced T helper 17 (TH17) cell-stimulating pathway correlating with improved survival 5.

To further evaluate the role of TLS in tumor progression, the relationship between TLS expression and clinicopathological characteristics in tumor patients were also analyzed and no significant association was found among TLS expression, age, gender, grade, ki67 expressions, N stage and T stage. However, in the subgroup of colorectal cancer and breast cancer, patient with lower N stage and clinical grade have higher expression of TLS in tumor tissue. Surprisingly, TLS expression is also correlated with the development of tumor size as indicated by the high TLS expression implies small tumor size especially in liver cancer and gastric tumor. Previous study has shown that the density of TLS plays an important role in the control of tumor growth in the elimination and equilibrium phase 32. Our study also found that high TLS expression had a positive correlation with infiltration of TILs in tumor tissues, which might be associated with the TLS function. Previous studies have revealed the function of TLS to recruit TILs, such as T cell, B cell, DC cell, through HEV or some chemokines, which could improve its antitumor immunity 53. As the part of TLS, HEV also can recruit TILs to defeat tumor cells by activating the nuclear factor κB (NF-κB) signaling pathway 54. In addition, TILs have some connection with the checkpoint blockade immunotherapy 55. That might mean that TLS plays an important role in immunotherapy. In our subgroup analysis, we found that in breast cancer patients, high TLS expression was associated with favorable clinicopathological characteristics (lower N stage, lower clinical grade and higher infiltration of TILs), indicating positive clinical outcome. However, there is no relationship between TLS expression and OS and DFS in the breast cancer subgroup. Therefore, further investigation is still required to explore the underlying mechanism. In addition, we also found that high TLS expression was related to a lower T stage, N stage and clinical grade in colorectal cancer. Previous study statistics showed that in colorectal cancer, increasing T stage was associated with a steady increase in rates of local recurrence. Meanwhile, the results of N stage also show that the increasing N stage was associated with the rising rate of 5-Year local recurrence 56. Therefore, low T stage, N stage and clinical grade were associated with low relapse rate in colorectal cancer patients with high TLS expression. Furthermore, high TLS expression has many immune pathways that enhance anti-tumor efficacy, which may be an important factor in reducing relapse in patients. Consequently, the relapse rate is lower in colorectal cancer patients with high TLS expression. Therefore, further research is needed to figure out the function of TLS in the treatment of cancer.

There are several limitations to our meta-analysis. First of all, some survival statistics calculated from the survival curve using Engauge Digitizer might have a certain degree of deviation even though the data were extracted very carefully. Secondly, the amount of research done in the subgroup analysis of OS, DFS, RFS, relapse rate and the type of tumors were not enough, to some extent, which could affect our understanding of the role of TLS. Finally, there are some studies that detected the TLS using different parts of it, for instance, B cells, T cells and HEV, which may cause some biases in our results.

## Conclusion

Despite the above-mentioned limitations, we have come to a conclusion that high TLS expression in the tumor is correlated with better OS in the oral squamous cell carcinoma and gastric tumor, better RFS and lower relapse rate. Meanwhile, TLS expression is related to tumor-infiltrating lymphocytes level and tumor size. In the subgroup of breast and colorectal cancer, high levels of TLS are overexpressed in cancer of low clinical grade and N stage. In summary, high expression of TLS is a potential prognostic marker in clinic for the assessment of patient survival and recurrence and its role in tumor immunotherapy is worth investigating in the future.

## Supplementary Material

Supplementary figures.

## Figures and Tables

**Table 1:** Characteristics of included studies.

First author	Year	Country	Types of tumor	Stage	Sample size	Laboratory method	Cut-off criteria	Estimate criteria	Follow-up time	Survival analysis	NOS score
Zhao Y.	2020	China	ESCC	I	593	HE	positive and negative	TLS	Median:42months (1 - 102 months)	OS	7
Zhang W.H.	2020	China	PDC	G1-G2	307	IHC	presence and the location of TLS	B cell, FDC, T cell	RFS: median: 39 months (1.5 - 95.0 months) OS: 58 months (10.0 - 96.0 months).	OS, RFS	9
Yamaguchi Y.	2020	Japan	CRC	II-III	67	IHC	number of TLS	TLS	Median:42.9 months (22.5 - 73.4 months)	Relapse	8
Lin Q.	2020	China	GT	NR	187	IHC	positive and negative	TILs	more than 100 months	OS, TTR	8
Li H.	2020	China	HCC	I-IV	462	IHC	positive and negative	TILs	Median:61.3 months (1.5 - 119.4 months)	OS, RFS	8
Yoshihito Y.	2020	Japan	GT	I-IV	226	IHC	percentage area (3%)	B cell, FDC, T cell, HEV	more than 80 months	DFS	7
He W.	2020	China	GT	I-III	1033	IHC/HE	positive and negative	HEVFDC	more than 100 months	OS	8
Li Q.	2020	China	OSCC	I-IV	168	IHC/HE	positive and negative	HEV, Immune cell	5 years	OS, RFS	8
Chao X.	2020	China	BC	NR	60	IHC	positive and negative	B cell, FDC, T cell HEV	Median:48months (22 - 163 months)	OS	8
Li K.	2020	China	OSCC	NR	65	IHC/HE	locations and counts of TLS (n = 4)	B cell, FDC, T cell HEV	Median:44 months (1 - 83 months)	OS, DFS	8
Sofopoulos M.	2019	Greece	BC	NR	112	IHC	locations and counts of TLS	HEV, Immune cell	0 -10 years	OS, DFS	7
Lee M.	2019	Korea	BC	I-V	335	IHC/HE	positive and negative	HEV, Immune cell	NR	OS	8
Kuwabara S.	2019	Japan	PDC	I-IV	47	IHC/HC	area of TLS	HEV, Immune cell	Median: 749.5 days	OS	8
Calderaro J.	2019	France	HCC	BCLC stage B-C	498	HE	positive and negative	TLS	0 -24 months	Relapse	8
Trajkovski G.	2018	Yugoslavia	CRC	I-IV	103	IHC	positive and negative	TILs	NR	NR	6
Posch F.	2018	Austria	CRC	II-III	109	IF	number of TLS	TLS	0 -36 months	Relapse	7
Song I. H.	2017	Korea	BC	I-IV	108	IHC	positive and negative	HEV	Median:34.9 months (12.0 - 55.8 months)	DFS	9
Liu X.	2017	China	BC	I-IV	245	HE	within 5 mm TLS	TLS	Median:78 months (1 - 134 months)	DFS, OS	8
Buisseret L.	2017	Canada	BC	I-III	125	IHC	positive and negative	TLS	NR	NR	6
Schweiger T.	2016	Vienna	CRC	I-IV	57	IHC	positive and negative	TLS	more than 80 months	OS, RFS	9
Lee H. J.	2016	Korea	BC	I-III	769	IHC	positive and negative	HEV, TILs	more than 100 months	OS, DFS	9
Silina K.	2015	Switzerland	LC	NA	87	IHC	positive and negative	TLS	5 years	DFS	6
Hiraoka N.	2015	Japan	PDC	I-IV	308	IHC	positive and negative	TLS	Median:17.6 months (2.6 - 201 months)	OS, DFS	9
Figenschau S. L.	2015	Norway	BC	I-III	290	IHC	positive and negative	TLS	NR	NR	6
Di Caro G.	2014	Italy	CRC	II-III	185	IHC	percentage area (2.68%)	TLS, TILs, HEV	Median: 4.71 years	Relapse	9
BehrD. S.	2014	Germany	Merkel cell carcinomas	I-IV	21	IHC	positive and negative	Immune cell	NR	OS, DFS	7
Anna	2014	Norway	OSCC	I-IV	80	IHC	within 100 um TLS	B cell, FDC, T cell, HEV	more than 100 months	DSS	8

**Abbreviations**: ESCC: esophageal squamous cell carcinoma; CRC: colorectal cancer; GT: gastric tumors; HCC: hepatocellular carcinoma; BC: breast cancer; PDC: pancreatic ductal carcinoma; LC: lung cancer; IHC: immunohistochemistry; H&E: hematoxylin-eosin staining; NOS: the Newcastle-Ottawa Scale; IF: immunofluorescence; OS: overall survival; RFS: relapse-free survival; DFS: disease-free survival; DSS: disease-specific survival; TLS: tertiary lymphoid structure; TILs: tumor-infiltrating lymphocytes; FDC: follicular dendritic cells; HEV: high endothelial venules.

**Table 2:** Association between TLS expression and clinicopathological characteristics of tumor patients.

Clinicopathological parameter	Studies (n)	Patients (n)	OR (95% CI)	P-value	Heterogeneity		
					I²%	P-value	Model
Age<60 vs. >60 years	9	2593	0.91(0.76-1.11)	0.36	18	0.28	Fixed
gastric tumor	2	1101	1.17(0.85-1.60)	0.33	45	0.18	
breast cancer	2	407	0.98(0.61-1.59)	0.94	0	0.42	
oral squamous cell carcinoma	2	248	0.84(0.47-1.50)	0.55	0	0.8	
pancreatic ductal carcinoma	2	343	0.80(0.44-1.43)	0.44	59	0.12	
other tumors	1	494	0.69(0.48-1.00)	0.05			
Gender (male vs. female)	9	1772	1.11(0.87-1.41)	0.40	16	0.30	Fixed
hepatocellular carcinoma	2	894	1.11(0.76-1.60)	0.6	63	0.1	
oral squamous cell carcinoma	2	248	1.27(0.68-2.34)	0.45	0	0.75	
pancreatic ductal carcinoma	2	355	1.19(0.68-2.07)	0.55	27	0.24	
other tumors	3	275	0.97(0.58-1.61)	0.89	64	0.06	
Tumor size (small vs. large)	6	2555	1.52(1.27-1.81)	<0.00001	48	0.09	Fixed
hepatocellular carcinoma	2	895	1.55(1.17-2.04)	0.002	0	0.49	
gastric cancer	2	1101	1.48(1.16-1.89)	0.002	86	0.0007	
other tumors	2	559	1.65(0.89-3.05)	0.11	46	0.17	
ki67 expressions (low vs. high)	3	509	0.71(0.29-1.75)	0.46	79	0.009	Random
Tumor-infiltrating lymphocytes level (TILs) (low vs. high)	3	635	0.15(0.10-0.21)	<0.00001	44	0.17	Fixed
breast cancer	3	635	0.15(0.10-0.22)	<0.00002	44	0.17	
T stage (T1-T2 vs. T3-T4)	8	1236	1.42(0.72-2.81)	0.31	55	0.03	Random
colorectal cancer	2	170	1.68(0.14-19.58)	0.68	44	0.18	
oral squamous cell carcinoma	2	237	1.48(0.16-13.54)	0.73	85	0.01	
pancreatic ductal carcinoma	2	355	3.51(1.06-11.63)	0.04	0	0.52	
other tumors	2	474	0.93(0.41-2.11)	0.87	44	0.18	
N stage (N0 vs. >N0)	9	1510	0.95(0.62-1.45)	0.81	60	0.01	Random
colorectal cancer	2	170	4.48(1.56-12.85)	0.005	0	0.99	
breast cancer	2	527	0.64(0.44-0.92)	0.02	0	0.77	
oral squamous cell carcinoma	2	232	0.90(0.16-4.89)	0.9	79	0.03	
pancreatic ductal carcinoma	2	355	0.97(0.40-2.34)	0.94	43	0.19	
other tumors	1	226	0.94(0.56-1.59)	0.83			
M stage (M0 vs. M1)	3	430	0.49(0.17-1.38)	0.18	0	0.81	Fixed
pancreatic ductal carcinoma	2	355	0.46(0.15-1.40)	0.17	0	0.60	
other tumors	1	75	0.91(0.04-23.43)	0.96			
Grade (I-II vs. III-IV)	11	2608	0.89(0.54-1.46)	0.64	76	<0.00001	Random
colorectal cancer	2	170	8.64(2.48-30.09)	0.0007	0	0.47	
breast cancer	3	620	0.31(0.21-0.47)	<0.00001	0	0.38	
gastric tumor	2	1140	0.87(0.69-1.10)	0.26	0	0.91	
pancreatic ductal carcinoma	2	355	2.22(0.62-7.92)	0.22	0	0.76	
other tumors	2	323	1.02(0.60-1.75)	0.93	0	0.76	

**Abbreviations:** vs: versus; T: primary tumor range; N: lymph node; M: distance metastasis; OR: odds ratios.

## Abbreviation

ESCC: esophageal squamous cell carcinoma
CRC: colorectal cancer
GT: gastric tumors
HCC: hepatocellular carcinoma
BC: breast cancer
PDC: pancreatic ductal carcinoma
LC: lung cancer
IHC: immunohistochemistry
IF: immunofluorescence
H&E: hematoxylin-eosin staining
NOS: the Newcastle-Ottawa Scale
OS: overall survival
DFS: disease-free survival
RFS: relapse-free survival
TILs: tumor infiltrating lymphocytes
TLS: tertiary lymphoid structure
HEV: high endothelial venules
TIME: tumor immune microenvironment
FRC: fibroblastic reticular cells
FDCs: follicular dendritic cells
MeSH: Medical Subject Headings
HR: hazard ratio
OR: odds ratios
