# Supplementary material for: Relationship between Tertiary Lymphoid Structure and the Prognosis and Clinicopathologic Characteristics in Solid Tumors
Source: Int J Med Sci. 2021 Apr 7;18(11):2327–38. doi: 10.7150/ijms.56347 (PMC8100653; doi:10.7150/ijms.56347)
Supplement: Supplementary file 1 — Supplementary figures. [file ijmsv18p2327s1.pdf]

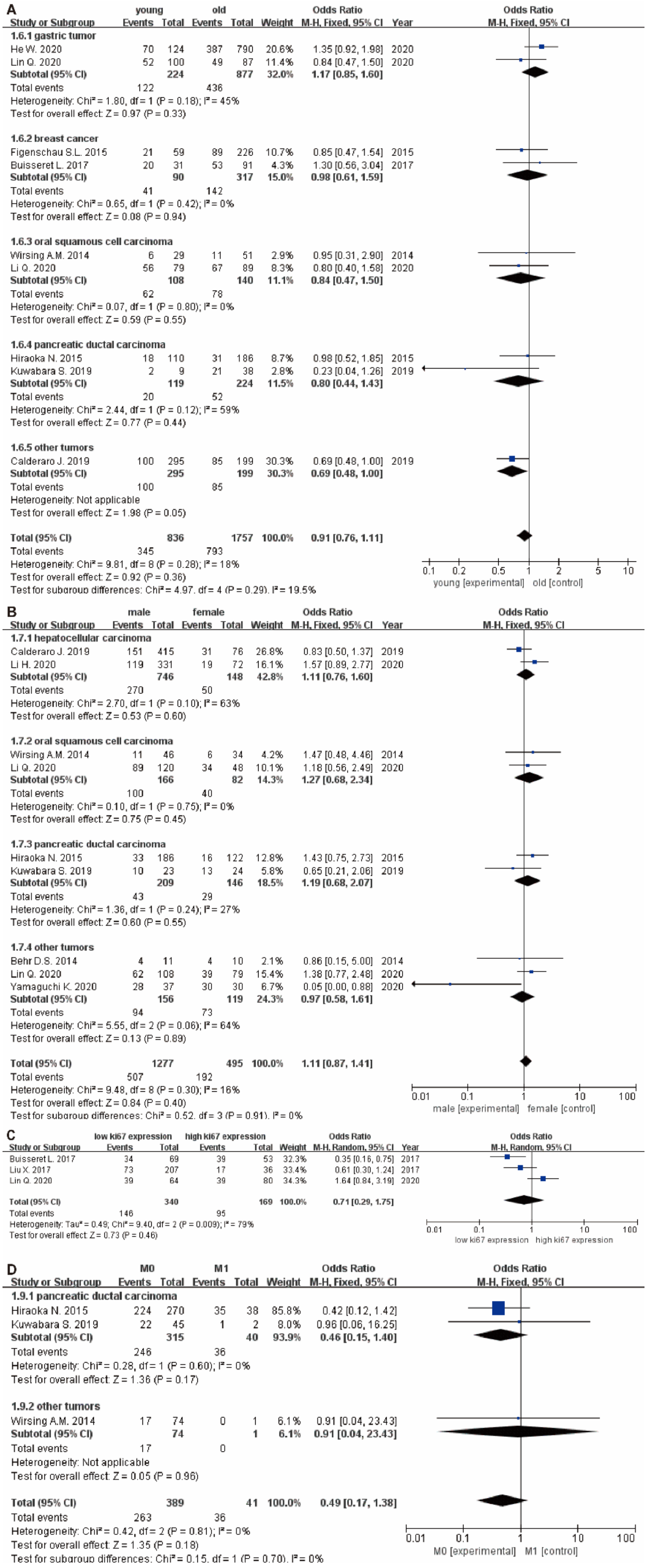

A
young
old
Odds Ratio
Odds Ratio
Study or Subgroup Events Total Events Total Weight M-H, Fixed, 95% CI Year
M-H, Fixed, 95% CI
1.6.1 gastric tumor
He W. 2020 70 124 387 790 20.6% 1.35 [0.92, 1.98] 2020
Lin Q. 2020 52 100 49 87 11.4% 0.84 [0.47, 1.50] 2020
Subtotal (95% CI) 224 877 32.0% 1.17 [0.85, 1.60]
Total events 122 436
Heterogeneity: Chi² = 1.80, df = 1 (P = 0.18); I² = 45%
Test for overall effect: Z = 0.97 (P = 0.33)
1.6.2 breast cancer
Figenschau S.L. 2015 21 59 89 226 10.7% 0.85 [0.47, 1.54] 2015
Buisseret L. 2017 20 31 53 91 4.3% 1.30 [0.56, 3.04] 2017
Subtotal (95% CI) 90 317 15.0% 0.98 [0.61, 1.59]
Total events 41 142
Heterogeneity: Chi² = 0.65, df = 1 (P = 0.42); I² = 0%
Test for overall effect: Z = 0.08 (P = 0.94)
1.6.3 oral squamous cell carcinoma
Wirsing A.M. 2014 6 29 11 51 2.9% 0.95 [0.31, 2.90] 2014
Li Q. 2020 56 79 67 89 8.3% 0.80 [0.40, 1.58] 2020
Subtotal (95% CI) 108 140 11.1% 0.84 [0.47, 1.50]
Total events 62 78
Heterogeneity: Chi² = 0.07, df = 1 (P = 0.80); I² = 0%
Test for overall effect: Z = 0.59 (P = 0.55)
1.6.4 pancreatic ductal carcinoma
Hiraoka N. 2015 18 110 31 186 8.7% 0.98 [0.52, 1.85] 2015
Kuwabara S. 2019 2 9 21 38 2.8% 0.23 [0.04, 1.26] 2019
Subtotal (95% CI) 119 224 11.5% 0.80 [0.44, 1.43]
Total events 20 52
Heterogeneity: Chi² = 2.44, df = 1 (P = 0.12); I² = 59%
Test for overall effect: Z = 0.77 (P = 0.44)
1.6.5 other tumors
Calderaro J. 2019 100 295 85 199 30.3% 0.69 [0.48, 1.00] 2019
Subtotal (95% CI) 295 199 30.3% 0.69 [0.48, 1.00]
Total events 100 85
Heterogeneity: Not applicable
Test for overall effect: Z = 1.98 (P = 0.05)
Total (95% CI) 836 1757 100.0% 0.91 [0.76, 1.11]
Total events 345 793
Heterogeneity: Chi² = 9.81, df = 8 (P = 0.28); I² = 18%
Test for overall effect: Z = 0.92 (P = 0.36)
Test for subgroup differences: Chi² = 4.97, df = 4 (P = 0.29), I² = 19.5%
0.1 0.2 0.5 1 2 5 10
young [experimental] old [control]
B
male
female
Odds Ratio
Odds Ratio
Study or Subgroup Events Total Events Total Weight M-H, Fixed, 95% CI Year
M-H, Fixed, 95% CI
1.7.1 hepatocellular carcinoma
Calderaro J. 2019 151 415 31 76 26.8% 0.83 [0.50, 1.37] 2019
Li H. 2020 119 331 19 72 16.1% 1.57 [0.89, 2.77] 2020
Subtotal (95% CI) 746 148 42.8% 1.11 [0.76, 1.60]
Total events 270 50
Heterogeneity: Chi² = 2.70, df = 1 (P = 0.10); I² = 63%
Test for overall effect: Z = 0.53 (P = 0.60)
1.7.2 oral squamous cell carcinoma
Wirsing A.M. 2014 11 46 6 34 4.2% 1.47 [0.48, 4.46] 2014
Li Q. 2020 89 120 34 48 10.1% 1.18 [0.56, 2.49] 2020
Subtotal (95% CI) 166 82 14.3% 1.27 [0.68, 2.34]
Total events 100 40
Heterogeneity: Chi² = 0.10, df = 1 (P = 0.75); I² = 0%
Test for overall effect: Z = 0.75 (P = 0.45)
1.7.3 pancreatic ductal carcinoma
Hiraoka N. 2015 33 186 16 122 12.8% 1.43 [0.75, 2.73] 2015
Kuwabara S. 2019 10 23 13 24 5.8% 0.65 [0.21, 2.06] 2019
Subtotal (95% CI) 209 146 18.5% 1.19 [0.68, 2.07]
Total events 43 29
Heterogeneity: Chi² = 1.36, df = 1 (P = 0.24); I² = 27%
Test for overall effect: Z = 0.60 (P = 0.55)
1.7.4 other tumors
Behr D.S. 2014 4 11 4 10 2.1% 0.86 [0.15, 5.00] 2014
Lin Q. 2020 62 108 39 79 15.4% 1.38 [0.77, 2.48] 2020
Yamaguchi K. 2020 28 37 30 30 6.7% 0.05 [0.00, 0.88] 2020
Subtotal (95% CI) 156 119 24.3% 0.97 [0.58, 1.61]
Total events 94 73
Heterogeneity: Chi² = 5.55, df = 2 (P = 0.06); I² = 64%
Test for overall effect: Z = 0.13 (P = 0.89)
Total (95% CI) 1277 495 100.0% 1.11 [0.87, 1.41]
Total events 507 192
Heterogeneity: Chi² = 9.48, df = 8 (P = 0.30); I² = 16%
Test for overall effect: Z = 0.84 (P = 0.40)
Test for subgroup differences: Chi² = 0.52, df = 3 (P = 0.91), I² = 0%
0.01 0.1 1 10 100
male [experimental] female [control]
C
low ki67 expression
high ki67 expression
Odds Ratio
Odds Ratio
Study or Subgroup Events Total Events Total Weight M-H, Random, 95% CI Year
M-H, Random, 95% CI
Buisseret L. 2017 34 69 39 53 32.3% 0.35 [0.16, 0.75] 2017
Liu X. 2017 73 207 17 36 33.4% 0.61 [0.30, 1.24] 2017
Lin Q. 2020 39 64 39 80 34.3% 1.64 [0.84, 3.19] 2020
Total (95% CI) 340 169 100.0% 0.71 [0.29, 1.75]
Total events 146 95
Heterogeneity: Tau² = 0.49; Chi² = 9.40, df = 2 (P = 0.009); I² = 79%
Test for overall effect: Z = 0.73 (P = 0.46)
0.01 0.1 1 10 100
low ki67 expression high ki67 expression
D
M0
M1
Odds Ratio
Odds Ratio
Study or Subgroup Events Total Events Total Weight M-H, Fixed, 95% CI
M-H, Fixed, 95% CI
1.9.1 pancreatic ductal carcinoma
Hiraoka N. 2015 224 270 35 38 85.8% 0.42 [0.12, 1.42]
Kuwabara S. 2019 22 45 1 2 8.0% 0.96 [0.06, 16.25]
Subtotal (95% CI) 315 40 93.9% 0.46 [0.15, 1.40]
Total events 246 36
Heterogeneity: Chi² = 0.28, df = 1 (P = 0.60); I² = 0%
Test for overall effect: Z = 1.36 (P = 0.17)
1.9.2 other tumors
Wirsing A.M. 2014 17 74 0 1 6.1% 0.91 [0.04, 23.43]
Subtotal (95% CI) 74 1 6.1% 0.91 [0.04, 23.43]
Total events 17 0
Heterogeneity: Not applicable
Test for overall effect: Z = 0.05 (P = 0.96)
Total (95% CI) 389 41 100.0% 0.49 [0.17, 1.38]
Total events 263 36
Heterogeneity: Chi² = 0.42, df = 2 (P = 0.81); I² = 0%
Test for overall effect: Z = 1.35 (P = 0.18)
Test for subgroup differences: Chi² = 0.15, df = 1 (P = 0.70), I² = 0%
0.01 0.1 1 10 100
M0 [experimental] M1 [control]

Supplementary Figure 1. The forest plot of OR was assessed for association between TLS and clinicopathological features: (A) age; (B) gender; (C) ki67 expression; (D) M stage. Each result was shown by the OR with 95% CI.

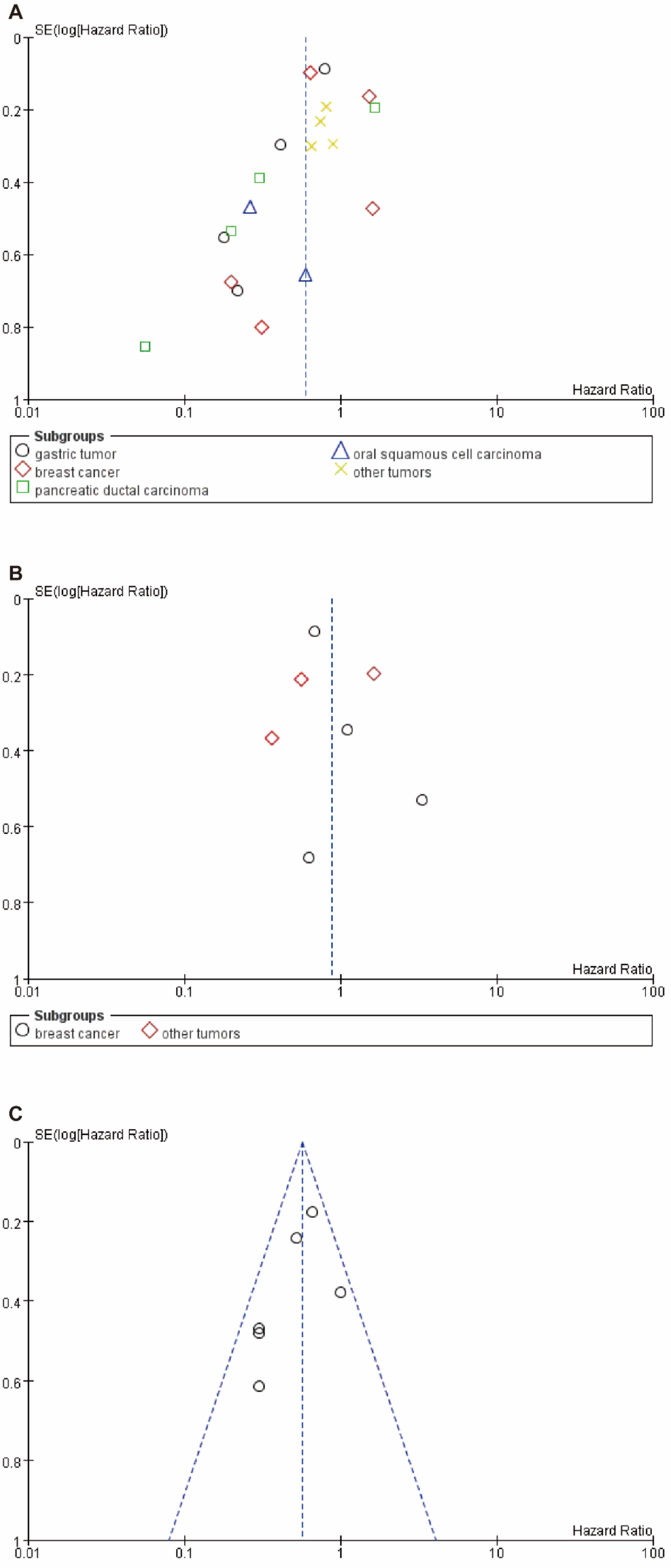
A
SE(log[Hazard Ratio])
0
0.2
0.4
0.6
0.8
1
Hazard Ratio
0.01
0.1
1
10
100
Subgroups
gastric tumor
breast cancer
pancreatic ductal carcinoma
oral squamous cell carcinoma
other tumors
B
SE(log[Hazard Ratio])
Hazard Ratio
Subgroups
breast cancer
other tumors
C
SE(log[Hazard Ratio])
Hazard Ratio

Supplementary Figure 2. Funnel plots of the association between TLS with survival: (A) OS; (B) DFS; (C) RFS.

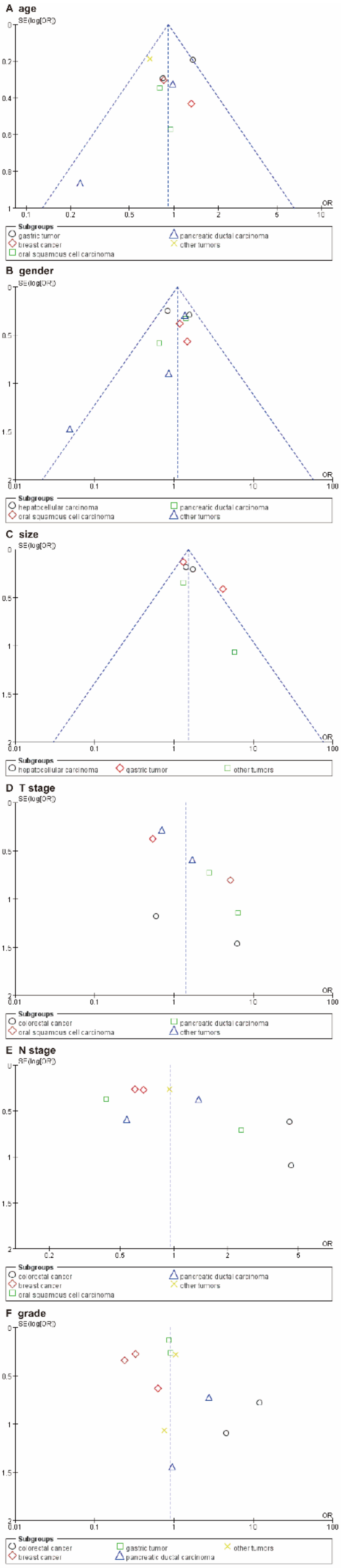
A age
SE(log[OR])
OR
Subgroups
gastric tumor
breast cancer
oral squamous cell carcinoma
pancreatic ductal carcinoma
other tumors
B gender
SE(log[OR])
OR
Subgroups
hepatocellular carcinoma
oral squamous cell carcinoma
pancreatic ductal carcinoma
other tumors
C size
SE(log[OR])
OR
Subgroups
hepatocellular carcinoma
gastric tumor
other tumors
D T stage
SE(log[OR])
OR
Subgroups
colorectal cancer
oral squamous cell carcinoma
pancreatic ductal carcinoma
other tumors
E N stage
SE(log[OR])
OR
Subgroups
colorectal cancer
breast cancer
oral squamous cell carcinoma
pancreatic ductal carcinoma
other tumors
F grade
SE(log[OR])
OR
Subgroups
colorectal cancer
breast cancer
gastric tumor
pancreatic ductal carcinoma
other tumors

Supplementary Figure 3. Funnel plots of the association between TLS expression with other clinicopathological characteristics: (A): age; (B): gender; (C): size; (D): T stage; (E): N stage; (F): grade.
